# Pleasant odors specifically promote a soothing autonomic response and brain–body coupling through respiratory modulation

**DOI:** 10.1038/s41598-025-20422-x

**Published:** 2025-10-17

**Authors:** Valentin Ghibaudo, Matthias Turrel, Jules Granget, Maëlys Souilhol, Samuel Garcia, Jane Plailly, Nathalie Buonviso

**Affiliations:** 1 Lyon Neuroscience Research Center (CRNL), Inserm U 1028, CNRS UMR 5292, Université Lyon1, 69675 Bron, France; 2 Neurophysiologie Respiratoire Expérimentale et Clinique UMRS 1158, Sorbonne Université, 75005 Paris, France

**Keywords:** Odors, Music, Pleasantness, Relaxation, Respiration, Cardiac activity, EEG, Brain-body, Autonomic, Respiration-related oscillations., Neuroscience, Cognitive neuroscience

## Abstract

Neuronal oscillations are tightly linked to physiological rhythms, yet little is known about how sensory stimuli influence their coupling. Here, we investigated whether pleasant odors and pleasant music modulate the coupling between respiration, autonomic activity, and brain oscillations. Using a within-subject design, we exposed participants to a personally pleasant odor and a personally pleasant music while recording respiratory and cardiac activity, feeling of relaxation, and respiration-locked EEG responses. Our results reveal that only odors induced a significant coupling between physiological and neural rhythms. Pleasant odors decreased respiratory rate, increased inspiratory volume, reduced heart rate, and enhanced heart rate variability (HRV). EEG analyses showed that this odor-induced respiratory slowing enhanced respiration-related brain activity in a temporo-parieto-central network, which is not observed with music. In contrast, music, regardless of tempo, increased subjective arousal without affecting autonomic or neural coupling. These findings suggest that pleasant odors engage an olfactomotor response, where an emotionally driven change in breathing rhythm enhances respiration-related neural oscillations. This mechanism may facilitate brain states associated with an increased feeling of relaxation and interoception, distinguishing odors as a unique sensory modality capable of promoting brain-body coupling. Our study highlights the potential of olfactory stimulation as a non-invasive tool for modulating brain rhythms and autonomic function.

**Supplementary Information:**

The online version contains supplementary material available at 10.1038/s41598-025-20422-x.

## Introduction

To promote positive emotions such as the feeling of relaxation, humans have long used breath control as a means of influencing the functioning of the autonomic nervous system and the subjective feeling of well-being^1^. Most empirically recognized methods for improving relaxation involve slowing down breathing, which reduces stress levels^1–3^. From a physiological perspective, slow breathing induces a shift from sympathetic dominance to a net increase in parasympathetic tone, resulting in a decreased heart rate^2^. From a neuronal perspective, studies on rodents have recently shown that respiration-related oscillations can be detected across large-scale brain networks when the animal breaths slowly and deeply, as during quiet wakefulness^4–6^. This effect is primarily due to the fact that slow nasal breathing optimally stimulates olfactory receptor neurons, which are sensitive to air pressure^7^. The resulting respiration-related activity is then propagated through the brain via the olfactory bulb, serving as a gateway for respiration-driven neural modulation in the brain. Cognitive factors, such as focused attention on breathing or volitional control, also increase coupling between brain activity and breath rhythm in the anterior cingulate, premotor, insular, and hippocampal cortices^8^. Interestingly, respiration can modulate brain activity in areas involved in emotion (as well as various cognitive functions), in both rodents and humans^8–10^. Conversely, emotions modulate breathing. While anxiety and stress increase breathing frequency^11–13^, pleasant feelings slow breathing down^14^. This emotional influence on breathing is based on the strong reciprocal connections between amygdala nuclei and respiratory regions^15^. As proof, electrical stimulation of the amygdala has been shown to accelerate respiratory rate in animals^16^ or induce apnea in humans^17–19^.

Olfaction plays a central role in the close link between emotion and respiration, leading us to conceptualize this relationship as a triad: Respiration – Olfaction – Emotion. Due to the unique connectivity between olfactory and limbic structures, as the olfactory bulb projects directly to limbic areas^20–22^, odors are primarily experienced through emotions^23,24^. Among them, pleasant odors hold a special status. Indeed, they slow down respiration rate and cardiac activity^25,26^ while unpleasant ones trigger rapid and superficial breathing^14^. Similarly, only pleasant odors (compared to unpleasant ones) can enhance mood and reduce both anxiety and pain unpleasantness^27^. However, the concept of pleasant smell should be approached with caution, as it is highly individual-dependent, given that odor pleasantness varies drastically between people^28^.

While aromachology and olfactotherapy harness the power of pleasant odors to influence mood, physiology, and behavior^29,30^, little is known about their underlying mechanisms. In this paper, our aim was to test the hypothesis that a personally pleasant odor – by slowing down respiratory rate – should also slow down other body rhythms (cardiac activity), facilitate the occurrence of a respiration-related EEG activity, and enhance the subjective feeling of relaxation. Moreover, we hypothesized that the olfactory modality would be more effective than any other sensory modality in inducing such effects. We tested this assumption by comparing the effects of a personally pleasant odor with those of personally pleasant music, often described as modulating physiological state^29^, on psychological, physiological, and neuronal responses.

## Materials and methods

### Participants and ethics statement

Thirty participants (16 women, 14 men, aged 27.3 ± 9.5 years, range: 19–51 years) took part in the study and attended two sessions at the Lyon Neuroscience Research Center (Registration number DPO service: 2-20125). The inclusion criteria were: aged between 18 and 60 years, social security coverage, and willingness to participate in the study. The exclusion criteria were: pregnancy, labor, or breastfeeding, deprivation of liberty by judicial or administrative decision, and known cardiovascular, respiratory, olfactory, neurosensory, or psychiatric disorders. This study was approved on May 17, 2022, by the Ethics Committee named Comité de Protection des Personnes Ile De France 3 (ID RCB: 2021-A03077-34) in accordance with French regulations for biomedical experiments with healthy volunteers. All research involving human participants was conducted in accordance with the Declaration of Helsinki. Participants were informed clearly and fairly about the study, provided written informed consent, and were compensated for their participation.

### Data and software

#### Psychological measures

The psychological measure of relaxation was assessed using the question “How relaxed do you feel?” which participants rated on a 10 centimeters continuous paper scale ranging from “Not at all”, to “Completely”. Furthermore, psychological arousal was evaluated using the question “How aroused do you feel?” following the same process. Responses were normalized to a scale from 0 to 1.

#### Physiological measures

Respiratory signal was recorded through nasal cannulas connected to a pressure sensor (Sensortechnics GmbH, Puchheim, Germany) that captured nasal airflow variations. The cardiac signal was recorded using an electrocardiogram (ECG, Brain Products GmbH, Gilching, Germany) with three electrodes placed on the anterior surface of the right wrist, the left wrist, and in the lower abdominal region at the left iliac fossa. Both respiratory and ECG signals were acquired using a DC amplifier (actiCHamp Plus Brain Products GmbH, Gilching, Germany) at a 1000 Hz sampling rate.

#### Electroencephalographic activity

Electroencephalographic (EEG) activity was recorded from the scalp using the same amplifier and sampling frequency as for ECG and respiratory signals (see 2.2.2), *via* 32 active EEG electrodes positioned according to the international 10–20 system using actiCAP nap (actiCAP Brain Products GmbH, Gilching, Germany). The ground electrode was placed on the mid-frontal region. Impedances were kept below 50 kΩ by applying conductive gel to the scalp.

### Stimuli and apparatus

#### Odors

Ten complex and presumed pleasant odors were composed by a perfumer (Sevessence, Dardilly, France, SIRET: 75348599400036). They were named Blue Ocean, Cotton Flower, Olive Leaf, Oxygen, Peach Lavender, Rose, Spiced Orange Blossom, Spiced Wood, Spring Floral, Vanilla. Fifteen-milliliter brown vials were filled to three-quarters with polypropylene beads soaked in 1 mL of an odorant solution diluted in isopropyl myristate (Hyteck Aroma-Zone, Paris, France). The dilution levels were adjusted for each perfume to ensure they were not overly intense while remaining easily perceptible for at least one hour. Supplementary Table 1 lists the dilution ratios for each perfume. For delivery, the vials were held by a custom-made articulated arm, positioned 10 cm from the participants’ noses.

#### Music pieces

Ten music pieces were preselected from the favorite genres of the French population (Supplementary Table 2). These genres included Classical Music, Electronic, French Variety, Hard Rock, Jazz, Metal, Pop, Rap, and World Music (Raga was chosen). The selected pieces were transformed into instrumental versions using Audacity (version 3.5.1, https://www.audacityteam.org/), to limit potential cognitive artifacts caused by mentally reciting lyrics. Average tempo of each sample was extracted through FL Studio software (version 21, https://www.image-line.com/) and confirmed by an experienced musician.

### Procedure

**Fig. 1:**
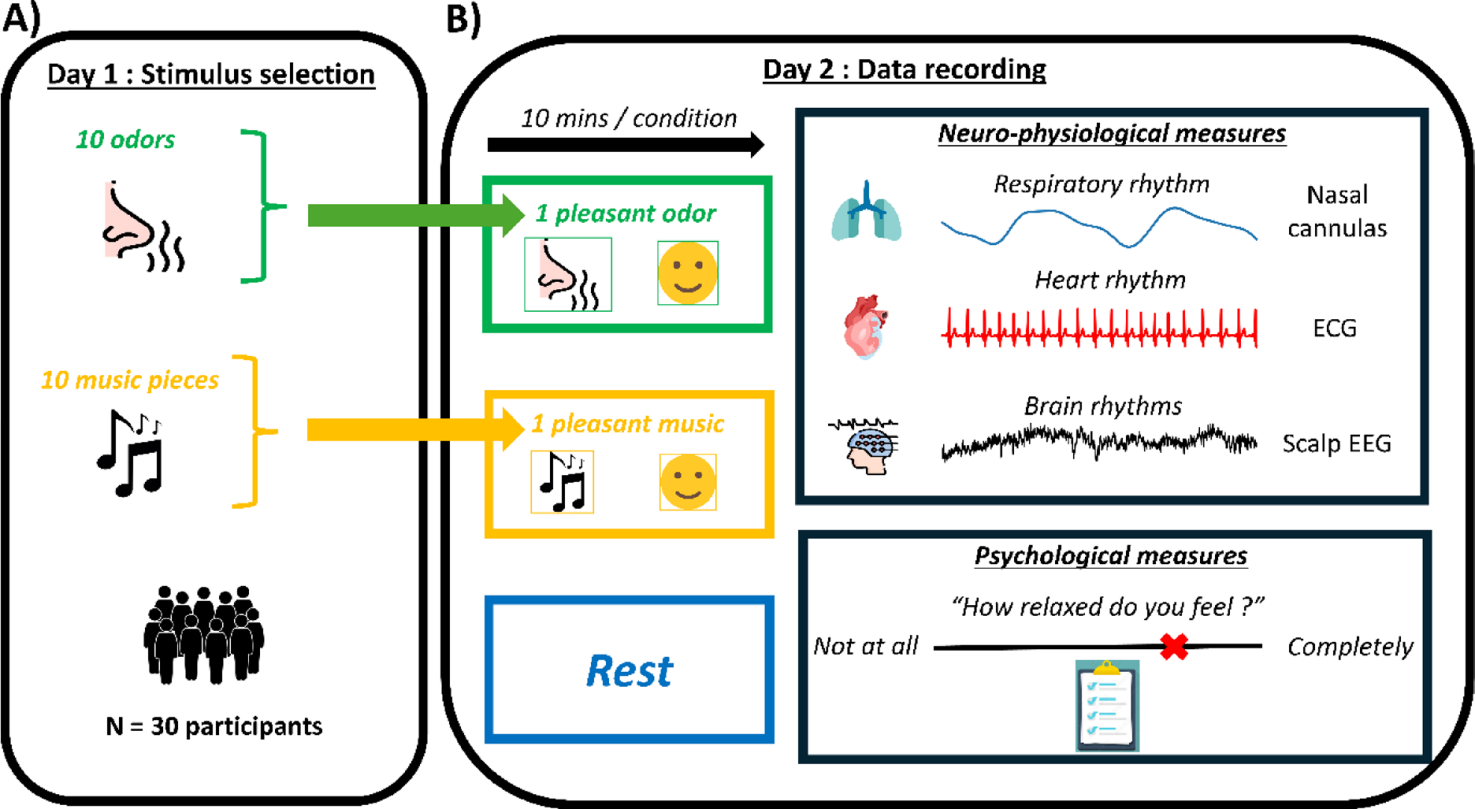
: Experimental design. Participants attended two sessions conducted on two different days: the first (**A**) aimed to select the most pleasant odor and the most pleasant music from a pre-selection of 10 that would be used in the second session (**B**), which aimed at recording physiological, neuronal and psychological data.

The experimental protocol consisted of two sessions conducted on two different days. The first session aimed to select the most pleasant odor and the most pleasant music to be used in the second session, which focused on recording physiological, neuronal and psychological data. The experimental protocol is schematically represented in Fig. 1.

#### Session 1: selection of individually most pleasant odor and music

In the first session, participants rated the odors and music pieces using two different methods: an absolute rating followed by a relative rating. The absolute rating involved evaluating the pleasantness of each stimulus independently on a continuous scale ranging from extremely unpleasant to extremely pleasant, with a central bar indicating neutrality. Based on this, the three highest-rated stimuli were selected for the relative rating. These were then rated in relation to one another (i.e., their ratings could be compared) based on their pleasantness. The odor and music with the highest pleasantness ratings were selected for Session 2. The obtained values were normalized on a scale from 0 to 1 (0 for “extremely unpleasant” and 1 for “extremely pleasant”).

#### Session 2: recording psychological, physiological and neuronal data in specific sensory environments

The second session took place one day after the first. The participants were asked to sit comfortably on a chair while keeping their eyes open. Once equipped with sensors (EEG, ECG, nasal cannulas), the participants were positioned in front of a desk, on which a mechanical arm was mounted to keep an odorant vial near the participant’s noses. A speaker (Essentielb SB70 Portable Speaker, Boulanger, Lille, France) was also placed on the desk one meter away. Three 10-minute recording blocks were conducted, during which participants were instructed to rest. First came a baseline block, during which no stimulus was presented. This was followed by two blocks of sensory stimulation (either odour or music), which were randomized and separated by a 15-minute break. After each block, participants were asked to evaluate, through paper questionnaires: (1) the perceived intensity of relaxation at that moment, and (2) the perceived arousal at that moment, both on a continuous scale ranging from “not at all” to “completely”. The values were normalized on a scale from 0 to 1.

### Data analysis

#### Physiological signals

All analysis was performed using a homemade Python toolbox^31^. We briefly describe the main processing steps here.

**Fig. 2:**
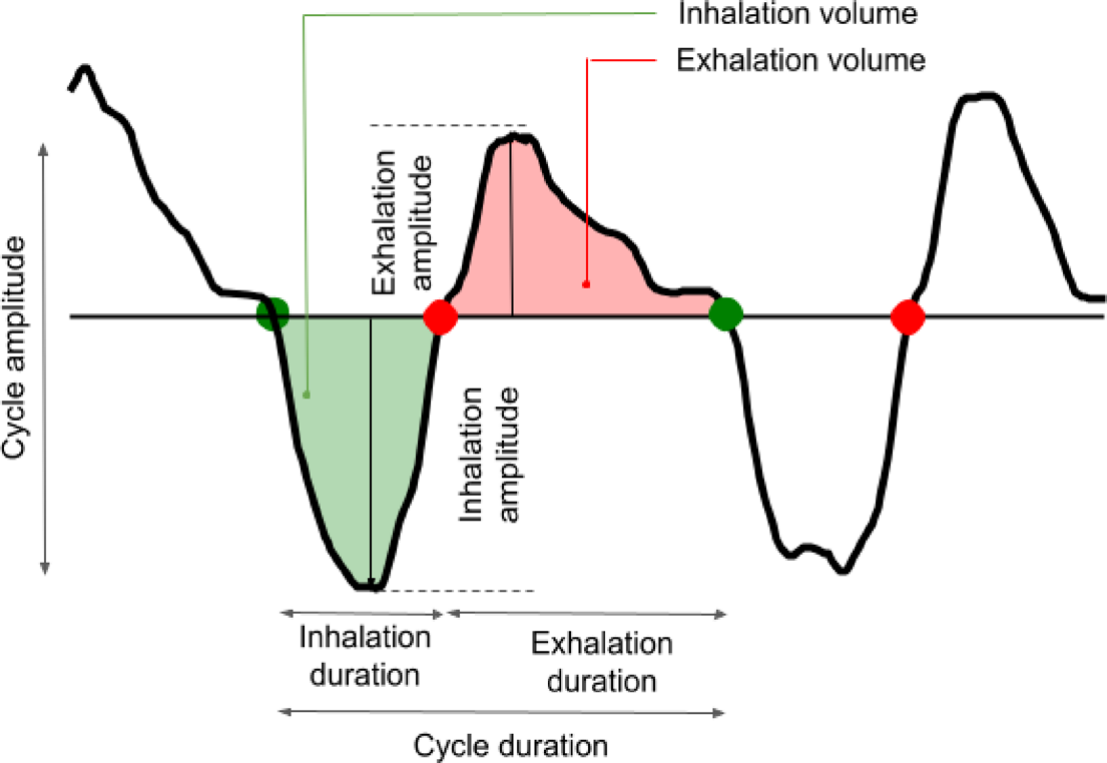
Detection of respiratory timestamps and calculation of respiratory features. The *physio* toolbox allows the processing of respiratory traces (black) enabling the detection of respiratory timestamps such as transitions from exhalation to inhalation (green dots) and from inhalation to exhalation (red dots) for each respiratory cycle. These detections enable the computation of respiratory features for each respiratory cycle, based on duration and amplitude of each phase (inspiration, expiration, cycle), which allows inferring the variations in the respiratory regime. Modified from Ghibaudo et al.^31^.

*Respiratory cycles* were extracted as follows. First, the respiratory signal was detrended and smoothed using a low-pass filter with a 7 Hz Bessel type. Then, Inspiration-Expiration transitions (IE) and Expiration-Inspiration transitions (EI) were detected when the respiratory signal rose and crossed its median level, and decayed and crossed its median level, respectively (see Fig. 2). Based on these detected timestamps, respiratory cycle characteristics, such as durations, amplitudes, and volumes, were computed cycle by cycle. Finally, aberrant respiratory cycles, resulting from faulty detection or artifacts, were statistically removed to ensure data accuracy and reliability, as described by Ghibaudo et al., ^31^.

*ECG signal* of each participant and experimental condition was first preprocessed by being centered and band-pass filtered using a Bessel filter (bandpass from 5 to 45 Hz). This step aimed to improve the signal-to-noise ratio to isolate the R peaks of the signal by removing other characteristic ECG waves considered as noise. Next, R peaks were detected with a minimum separation of 400 milliseconds. Then, R-R Intervals (RRI) were computed, representing the time duration between consecutive heartbeats. The median value of the RRIs was computed as a proxy for heart rate. The Median Absolute Deviation (MAD) of these computed RRI was used as a measure of Heart Rate Variability (HRV), as MAD is more robust to outliers than standard-deviation (SD)^32^.

#### EEG signals

##### Preprocessing

The signal recorded by each electrode was re-referenced to the common average. Slow drifts were removed through linear detrending, and the resulting traces were centered by subtracting the median value. The signal was band-stop filtered (50 Hz, Notch filter for line noise removal) and band-pass filtered (from 0.05 to 200 Hz, Butterworth type, order 6). Independent Component Analysis (ICA) was applied to exclude ocular artifact components (blinks and ocular-motor movements, characterized by high-amplitude phasic components, especially in the fronto-polar region). This process was performed using the MNE library, version 1.4.2^33^. Artifacted channels were manually removed. Finally, movement artifacts and the corresponding respiratory epochs were removed based on the criteria of large statistical deviations (> 4.5 median absolute deviations from median baseline level) of gamma power.

##### Measurement of respiration-related EEG activity

**Fig. 3:**
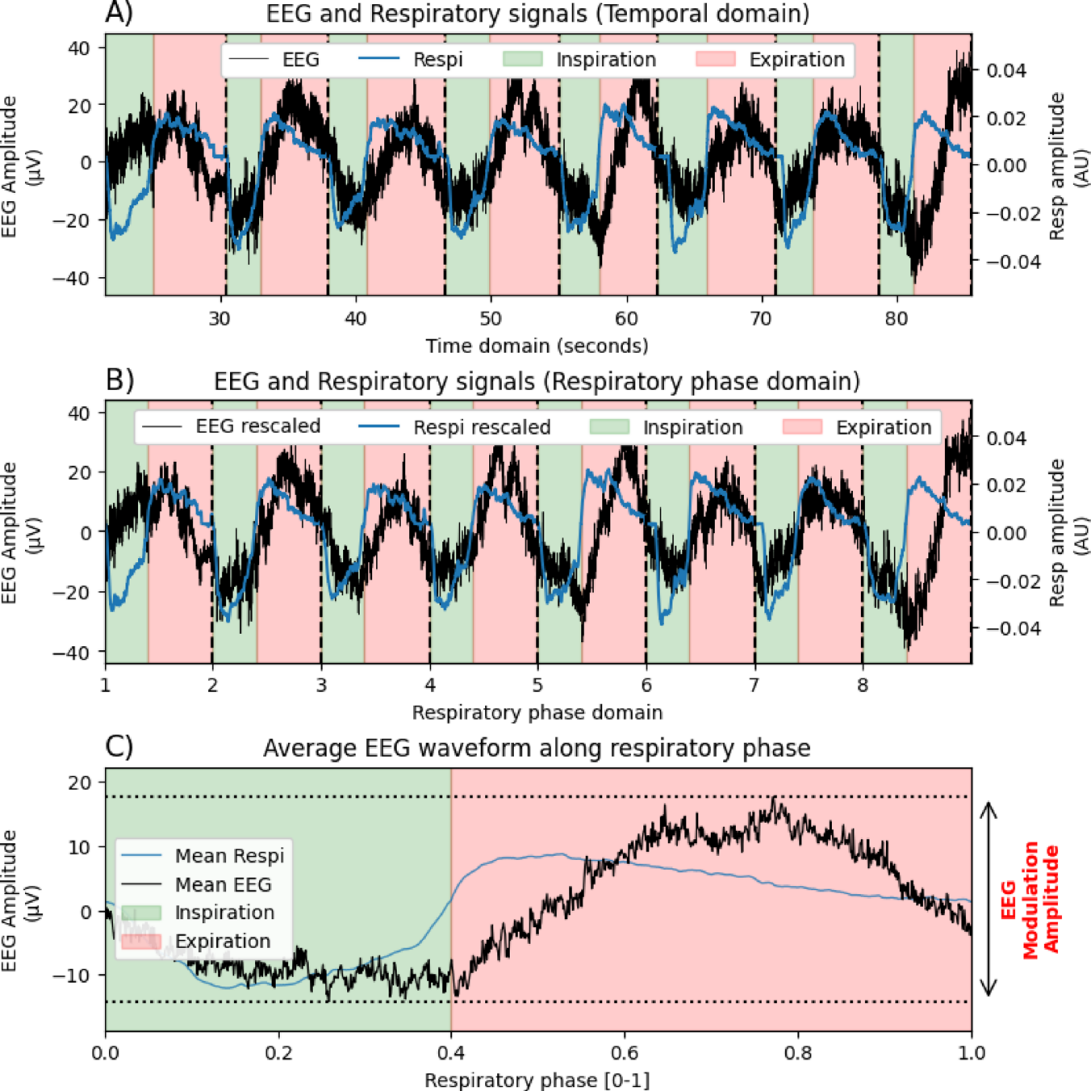
Pipeline of measurement of respiration-related EEG activity. (**A**) An example of an EEG time series is presented in black, and the corresponding respiratory time series (nasal airflow) is presented in blue. Inspiratory and expiratory epochs, deduced from respiratory feature detection, are shown in green and red spans, respectively. Black vertical dashed lines indicate the separation between two successive respiratory cycles. Note the variability in the duration of the eight successive respiratory epochs/cycles, which would prevent the averaging of respiratory-based EEG epochs. (**B**) Time series are rescaled into respiratory phase-based series through linear interpolation of traces between detected respiratory timestamps (IE and EI transitions). Therefore, each respiration-based EEG epoch has the same length and can be averaged across epochs/cycles. (**C**) The averaged EEG cyclical dynamic along the respiratory phase is displayed in black. The total amplitude of this trace can be computed (max–min) to extract the strength of modulation of EEG amplitude by respiration: “EEG Modulation Amplitude”. AU, arbitrary units; Respi, respiration.

The respiratory characteristics (IE points, EI points, inspiratory and expiratory amplitudes, and inspiratory, expiratory, and total volumes), extracted on a cycle-by-cycle basis, were utilized to achieve a cyclical deformation of the preprocessed brain signal based on the respiratory phase. This step was automated by the *deform_traces_to_cycle_template()* function coded in the physio toolbox^31^ which rescales the brain signal time basis based on the timestamps of respiratory phase transitions (EI and IE timestamps) through linear interpolation. This rescaled signal was segmented to provide one neural epoch per respiratory cycle with a common time vector (the respiratory phase) which was averaged to obtain the mean EEG signal along the respiratory cycle. Then, the strength of the modulation of EEG activity by respiration (“Respiration-related EEG activity”) was extracted by measuring the amplitude of mean EEG signal (maximum value – minimum value) for each electrode, experimental condition, and participant. This process is schematically represented in Fig. 3.

### Statistics

Except for cluster-based permutation statistics used for analyzing EEG results, we used statistical tests from *pingouin*^34^, a Python-coded toolbox dedicated to statistics, in order to compare the three experimental conditions: baseline, odor, and music. Data were considered significantly different if *p*-value was < 0.05.

#### Respiratory cycle duration, cycle volume, median RRI, HRV MAD, relaxation, arousal

Psychological and physiological metrics were obtained with a sample size of *N* = 30, repeatedly (within-participant design). We first assessed the sphericity and normality of the data, using Mauchly’s test and Shapiro-Wilk test, respectively. When data presented a violation of sphericity or normality, we used a non-parametric within-participant design test allowing for comparison of three conditions: the Friedman test. This was the case for all metrics except for Median RRI and HRV MAD, for which we used parametric within-participant design tests: repeated-measure analyses of variance (ANOVA). If the effect of experimental conditions was significantly different, we extended explorations by using two-sided pairwise *post-hoc* tests: *t*-tests or Wilcoxon test according to the sphericity and normality of data. *p*-values were adjusted for multiple comparisons with the Holm–Bonferroni method.

#### Respiration-related EEG activity

The EEG Modulation Amplitude was obtained for each participant, electrode, and experimental condition. Preserving the topographical relationship of the obtained values allowed for the computation of cluster-based permutation tests to compare music and odor values to those obtained in baseline. To do this, we used the *mne.stats.permutation_cluster_1samp_test()* function from the MNE toolbox^33^. This function allows for non-parametric cluster-level paired *t*-test, as described in Maris and Oostenveld^35^. We provided the function with a 30 (participants) * 32 (electrodes)-sized matrix with the difference between odor and baseline conditions. The same process was used to compare music to the baseline condition. The threshold parameter of the function was set to None (default), meaning that the cluster-forming threshold was chosen automatically based on a *p*-value of 0.05 for the given number of observations. The number of permutations was set to the default: 1024. The tail parameter was set to 0, meaning that the statistic is computed on both sides of the null distributions.

#### Correlations and regressions

Statistical interactions between metrics were explored through the computation of quantitative statistics with Pearson correlations and linear regressions using *scipy.stats.pearsonr* and *scipy.stats.linregress* functions from *SciPy* toolbox^36^.

## Results

### Pleasantness evaluation of the stimuli

The selected stimuli were all very pleasant (pleasantness > 0.5) with a pleasantness of 0.91 (± 0.07, [0.88, 0.93]; mean ± standard deviation, 95% confidence interval) for odors and of 0.93 (± 0.07, [0.90, 0.95]) for music. Pleasantness ratings were similar for odors and music (Wilcoxon test, W = 139, *p* = 0.15).

### Neither odor nor music influenced subjective feelings of relaxation; both affected subjective feeling of arousal

**Fig. 4:**
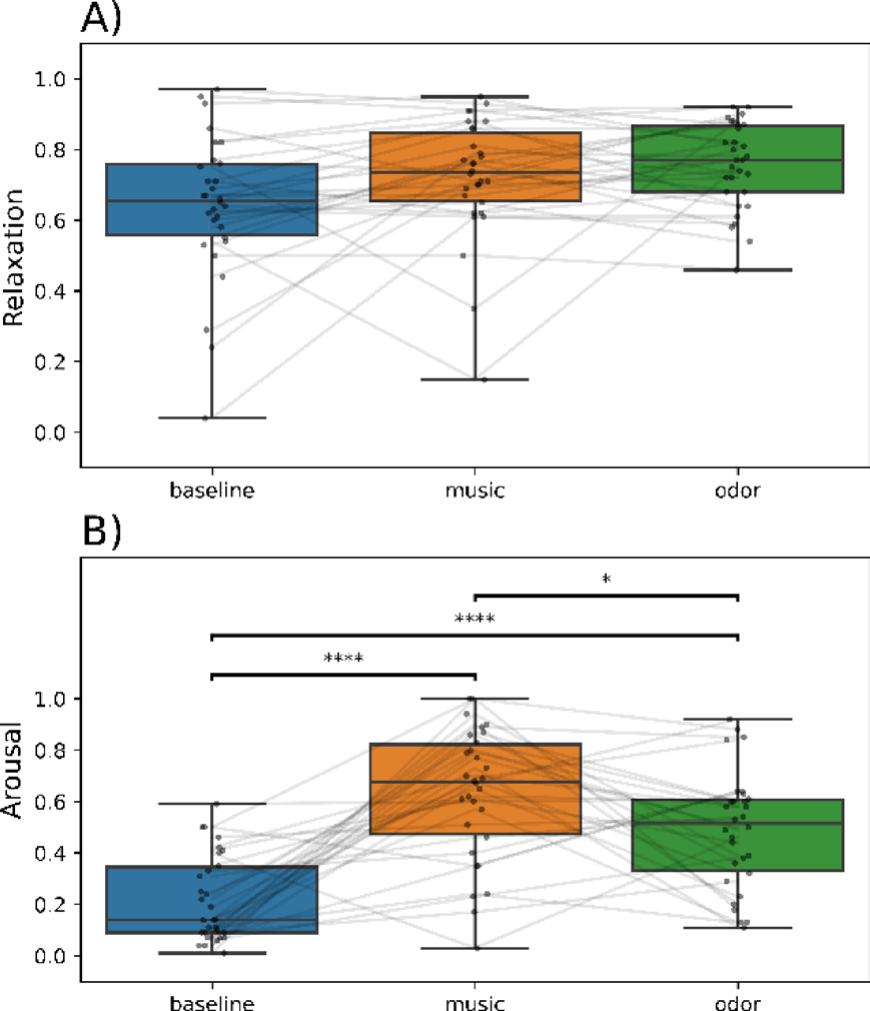
Effects of experimental conditions on measures of (**A**) relaxation and (**B**) arousal. The distribution of data is displayed as boxplots (minimum, first quartile, median, third quartile, and maximum). Individual data points are superimposed on the plots, with lines connecting those from the same participant. *, *p* < 0.05; ****, *p* < 0.0001.

As evidenced in Fig. 4A, the subjective feeling of relaxation was 0.64 (± 0.20, CI95: [0.56, 0.72]; mean ± standard deviation, CI95) after the baseline condition, 0.72 (± 0.17, [0.66, 0.78]) after the music condition, and 0.75 (± 0.12, [0.71, 0.80]) after the odor condition. Despite a tendency for both music and odor conditions to increase relaxation, no significant effect of experimental conditions on subjective relaxation was observed (W = 0.07, *p* = 0.12). Because the music selected by the participants had different tempi and because tempo plays an important role in inducing relaxation^31^, we analyzed the correlation between tempo and subjective relaxation (Supplementary Fig. 1). No significant relationship between tempo and subjective relaxation (Pearson correlation, *r* = 0.26, *p* = 0.17; *r*^2^ = 0.05, *p* = 0.24) was observed. Thus, neither odor nor music, regardless of its tempo, significantly influenced subjective feelings of relaxation.

As shown in Fig. 4B, subjective arousal increased both during the music and odor conditions. Subjective arousal significantly differed between experimental conditions (Friedman test, W = 0.58, *p* < 0.001) with both music (0.63 ± 0.26, [0.53, 0.73]) and odor (0.49 ± 0.23, [0.40, 0.57]) conditions significantly differing from baseline (0.22 ± 0.17, [0.16, 0.28], *p* < 0.0001). Therefore, both odor and music were perceived as arousing, even though this was more pronounced for music than for odor. Furthermore, no significant relationship between the tempo of music and subjective arousal was found (*r* = -0.005, *p* = 0.98, *r*^2^ = 0.001, *p* = 0.89; Supp. Fig. 1B).

### Pleasant smells, but not pleasant music, slow down body rhythms

#### Respiratory activity

For each experimental block and participant, a cycle-by-cycle computing (see Methods) allowed to measure the median respiratory cycle duration (1/frequency), the median inspiratory volume, median expiratory volume and total median cycle volume.

**Fig. 5:**
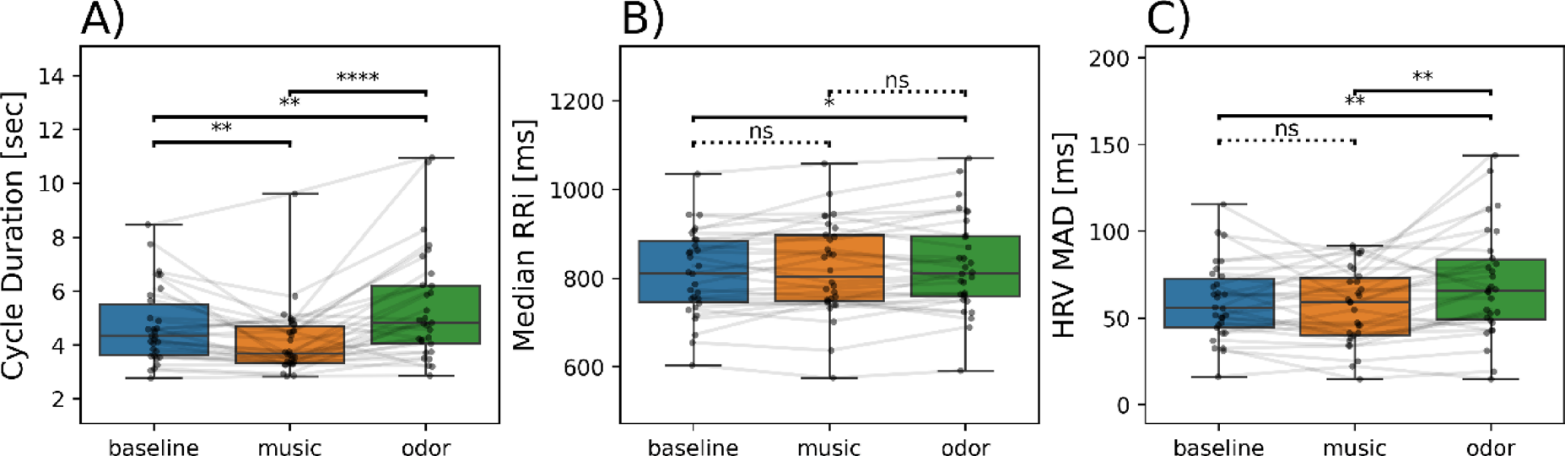
Effects of experimental conditions on (**A**) respiratory cycle duration, (**B**) median RRI from cardiac signal, and (**C**) HRV MAD from cardiac signal. The distribution of data is displayed as boxplots (minimum, first quartile, median, third quartile, and maximum). Individual data points are superimposed on the plots, with lines connecting those corresponding to the same participant. *, *p* < 0.05; **, *p* < 0.01; ***, *p* < 0.001. AU, arbitrary units.

Respiratory cycle durations were significantly different between conditions (Friedman test, W = 0.29, *p* < 0.001; Fig. 5A). Respiratory cycles were shorter in the music condition (4.14 ± 1.29 s, [3.66, 4.63]) and longer in the odor condition (5.41 ± 2.06 s, [4.64, 6.18]) than in the baseline condition (4.71 ± 1.43 s, [4.18, 5.24]); *p’s* < 0.01). A similar trend was observed for inhalation (Friedman test, W = 0.52, *p* < 0.001) and exhalation (Friedman test, W = 0.23, *p* < 0.001) durations, both of which were significantly different between conditions (not shown). Indeed, inhalation durations were shorter in the music condition (1.59 ± 0.52 s, [1.40, 1.79]) and longer in the odor condition (2.20 ± 0.93 s, [1.85; 2.55]) than in the baseline condition (1.81 ± 0.54 s, [1.61, 2.01]). Similarly, exhalation durations were shorter in the music condition (2.49 ± 0.83 s, [2.18, 2.80]) and longer in the odor condition (3.16 ± 1.24 s, [2.70, 3.62]) than in the baseline condition (2.86 ± 0.98 s, [2.49, 3.23]). No significant relationship between music tempo and respiratory cycle duration was shown (Spearman correlation test, *r* = 0.019, *p* = 0.92; *r*^2^ = 0.012, *p* = 0.57).

Overall, these results showed that pleasant music accelerated breathing, regardless of its tempo, while pleasant odors slowed it down.

#### Cardiac activity

As shown in Fig. 5B, the median RRI significantly differed between experimental conditions (*F*(2,58) = 4.19, *p* = 0.02), with a significantly higher median RRI in odor condition (830.18 ± 108.55 ms, [789.65, 870.71]) than in the baseline condition (811 ± 97.10 ms, [774.74, 847.26]; *p* < 0.05). No difference was observed between the music condition and the baseline condition (821.08 ± 106.35 ms, [781.37, 860.79]; *p* > 0.05). Tempo could theoretically influence heart rate by its rhythm. However, no significant correlation was found between median RRI and tempo (*r* = 0.205, *p* = 0.28; *r*^2^ = 0.03, *p* = 0.34; Supp. Fig. 1B). Thus, these results showed that only pleasant odors increased median RRI, i.e., decreased heart rate. In contrast, music did not affect median RRI, regardless of its tempo.

Median Absolute Deviation (MAD) of the computed RRI was used to extract Heart Rate Variability (HRV), serving as a proxy for vagal tone. HRV MAD (Fig. 5C) significantly differed between conditions (*F* (2,58) = 7.76, *p* = 0.001) with a significantly higher HRV MAD in the odor condition (69.56 ± 31.01 ms, [57.98, 81.14]) compared to baseline condition (59.55 ± 22 ms, [51.19, 67.91]; *p* < 0.01). No difference was observed between the music condition and the baseline condition (57.28 ± 21.98 s, [49.07, 65.49]; *p* > 0.05). Again, no significant correlation was found between HRV MAD and tempo (*r* = 0.02, *p* = 0.92; *r*^2^ = 0, *p* = 0.93; Supp. Fig. 1C, ). Thus, these results showed that only pleasant odors increased heart rate variability. In contrast, music did not affect HRV MAD, regardless of its tempo.

### Pleasant odors, but not pleasant music, promote a coupling between respiration and EEG activity

**Fig. 6:**
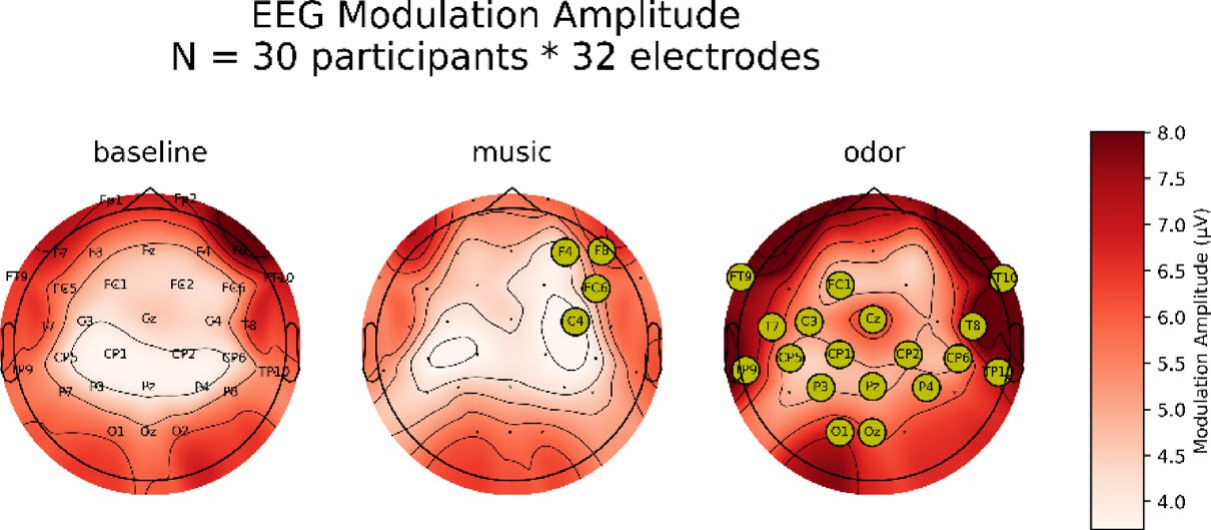
Respiration-related EEG activity induced by music and odors. The amplitude of modulation of the EEG signal by the respiratory phase was obtained for each participant, electrode, and experimental condition. Averaged values are topographically represented for the three experimental blocks. EEG modulation by the respiratory phase was significantly increased compared to baseline conditions (*p* < 0.05) at electrodes marked by yellow dots.

As evidenced by the topographic representation (Fig. 6), the amplitude of respiration-related EEG activity significantly increased (*p* < 0.05) in a bilateral temporo-parieto-central pattern, but only during the odor condition. The music condition did not differ from baseline except for a few electrodes (F4, F8, FC6, C4).

We then tested whether this respiratory-related EEG modulation could be influenced by the tempo of the different musical pieces. However, cluster-based permutation tests comparing each condition of tempo condition to baseline condition did not reveal significant effect of music, regardless of tempo (Supplementary Fig. 2).

**Fig. 7:**
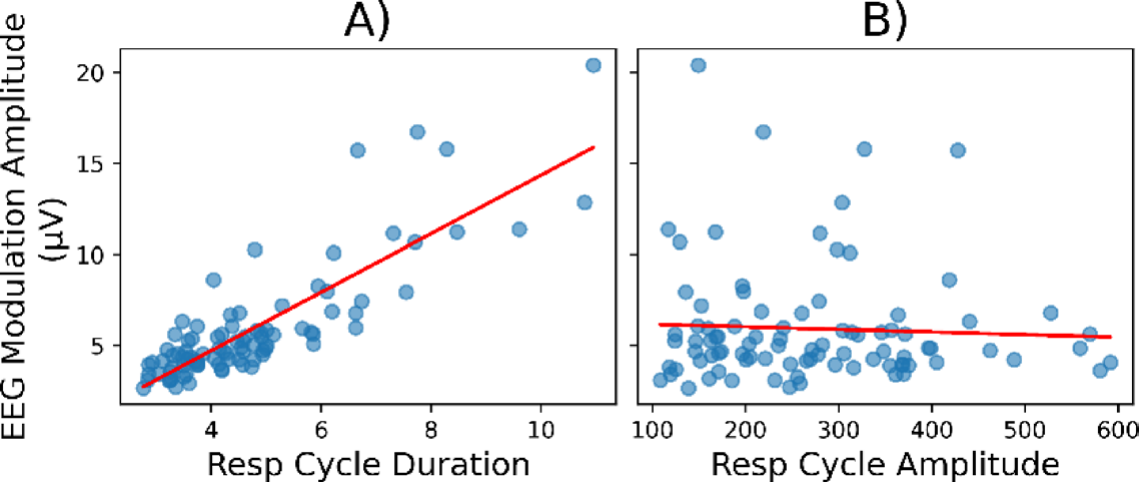
Correlation between respiratory parameters and respiration-related EEG activity. For each participant and experimental condition, the average respiration-related EEG amplitude is plotted against (**A**) the corresponding average respiratory cycle duration, (**B**) the corresponding average respiratory cycle amplitude. A red line represents the regression fit. Resp, respiratory.

Finally, we investigated a possible mechanistic relationship between respiratory cycle parameters and respiration-induced brain activity. Specifically, we examined the correlations between (A) respiratory cycle duration and (B) respiratory cycle volume with the amplitude of respiration-related EEG activity (Fig. 7). We found that the amplitude of respiration-related EEG activity was significantly correlated with respiratory cycle duration (Fig. 7A; *r* = 0.78, *p* < 0.0001) and, to a lesser extent, with respiratory cycle volume (Fig. 7B; *r* = 0.38, *p* < 0.001). The weaker correlation with volume compared to duration can be explained by the fact that amplitude of respiratory-modulated EEG activity was not correlated with respiratory cycle amplitude (Fig. 7C; *r* = -0.007, *p* > 0.05). These data should be interpreted with caution because measuring respiratory volume and/or amplitude is limited using a pressure sensor.

Overall, these results suggest that pleasant odors, by slowing respiratory rate, induced a respiration-related activity in the brain. Conversely, pleasant music, which was not able to slow down respiratory rate regardless of its tempo, only elicited a decrease in respiration-related brain activity in a limited number of sites.

## Discussion

The aim of the present study was to investigate the key role of olfaction in slowing physiological and brain rhythms and in enhancing the feeling of relaxation by comparing the effects of pleasant odors and pleasant music from three complementary perspectives: psychological, physiological and neuronal. Psychologically, we found that both odor and music tended to improve subjective feelings of relaxation and had a significant effect in increasing arousal. Physiologically, we evidenced a soothing effect specific to odors: pleasant odors, but not pleasant music, slowed down respiration, increased inspiratory volume, decreased heart rate, and increased HRV. From the neuronal perspective, pleasant odors, but not pleasant music, are able to enhance respiration-related brain activity in a temporo-parieto-central network, due to the olfacto-motor act they trigger. Our study reveals that, rather than a general impact of stimulus pleasantness, the effects we observed are a unique contribution of odors. For the first time, we demonstrate that a personally pleasant odor, by modulating the olfactomotor response, is an optimal sensory stimulus to promote coupling between body and brain rhythms.

### The key role of olfactomotor response in Body-Brain Rhythm coupling induced by pleasant odors

Several research groups have already reported the potent effect of odors on breathing and autonomic responses^37,38^. In particular, pleasant odors, slow down breathing^39,40^ and cardiac frequency^25^. Interestingly, even an odor that is not inherently pleasant, can nevertheless slow breathing frequency and increase amplitude if it has been previously associated with a pleasant taste^41^. Furthermore, when a participant is instructed to mentally imagine a smell, the imagery of pleasant odors elicits larger sniffs than that of unpleasant odors^39^. In an episodic memory task, participants breathed more deeply and for a longer duration in response to pleasant odors than to unpleasant ones^42^. Similarly, the retrieval of a positive autobiographical memory triggered by a personally pleasant odor is associated with slower and deeper breathing compared to the perception of control odors evoking no memory^26^. Taken together, these findings strongly suggest that an odorant stimulus triggers an olfactomotor response, primarily characterized by breathing modification.

The modification of breathing patterns, specifically a slowdown, first triggers an autonomic response marked by an increase in heart rate variability. This phenomenon could be attributed to the well-known cardio-respiratory coupling, whose primary consequence is that a decrease in respiratory rate leads to an increase in the amplitude of respiratory sinus arrhythmia (RSA), recently renamed respiratory heart rate variability^43^. RSA is a form of cardio-respiratory coupling that manifests as variations in cardio-inhibitory vagal parasympathetic tone during the respiratory cycle^44–46^. This tone decreases during inhalation and increases during exhalation, leading to heart rate oscillations synchronized with respiration^44,47^. Studies have demonstrated that the amplitude of these oscillations increases as the respiratory rate slows down^48–50^. Thus, it is very highly probable that the HRV increase we observed in response to odor-induced respiratory slowdown is primary driven by an increase in RSA amplitude. Interestingly, some theories suggest a link between the withdrawal of RSA and heightened anxiety^51^, implying a possible association between enhanced RSA and a relaxed physiological state. However, these ideas remain highly debated.

We also demonstrated that an odor-induced increase in respiratory cycle duration (i.e. a slower breathing) led to a stronger respiratory drive of the brain. The respiratory rhythm has been observed in both rodents and humans to influence brain oscillations across various brain regions through the impact of breathing-related airflows on olfactory receptor cells^5,8,10,52–58^. We previously showed in rodents that a slow and deep respiratory pattern, characteristic of the resting state, optimally enhances the influence of respiration on brain activity^4,54^. Here, we extend this finding by showing that, in humans, a personally pleasant odor, through the olfactomotor response it provokes, can induce a respiration-related rhythm within a large temporo-parieto-central network. This slow brain rhythm offers sufficiently long temporal windows to recruit large neural assemblies, thereby enabling the coordination of long-distance networks, including those involved in the default mode activity of the brain^59–61^. This raises the possibility that olfactomotor responses triggered by pleasant odors could facilitate the engagement of the brain in its default mode^62^. Given that the default mode is characterized by long-distance cortical communication, it aligns with the idea that such widespread functional connectivity is a key parameter in evaluating states of consciousness. In line with this, the “global workspace” theory suggests that the exchange of information between brain regions—and their synchronization—serves as a marker of consciousness^63^. Supporting this framework, studies have shown that global signal synchronization, as measured through functional connectivity analysis, correlates with an increased feeling of consciousness^64–66^, whereas disruptions in functional connectivity are associated with disorders of consciousness^67,68^. Interestingly, brain rhythms are known to synchronize with breathing^8,10^, and slow oscillations induced by nasal breathing have been shown to enhance global connectivity and alter consciousness perception^1,54,69^. These findings suggest a potential mechanistic link between respiration- related brain oscillations and the beneficial effects of odor exposure.

Thus, our results could provide insight into the mechanisms underlying odor-induced clinical benefits, particularly through respiratory-driven global brain rhythm modulation. Furthermore, future studies could investigate the extent to which the sensation of “altered consciousness” contributes to feeling of relaxation, refining our understanding of behavioral differences between odor and music.

**Fig. 8:**
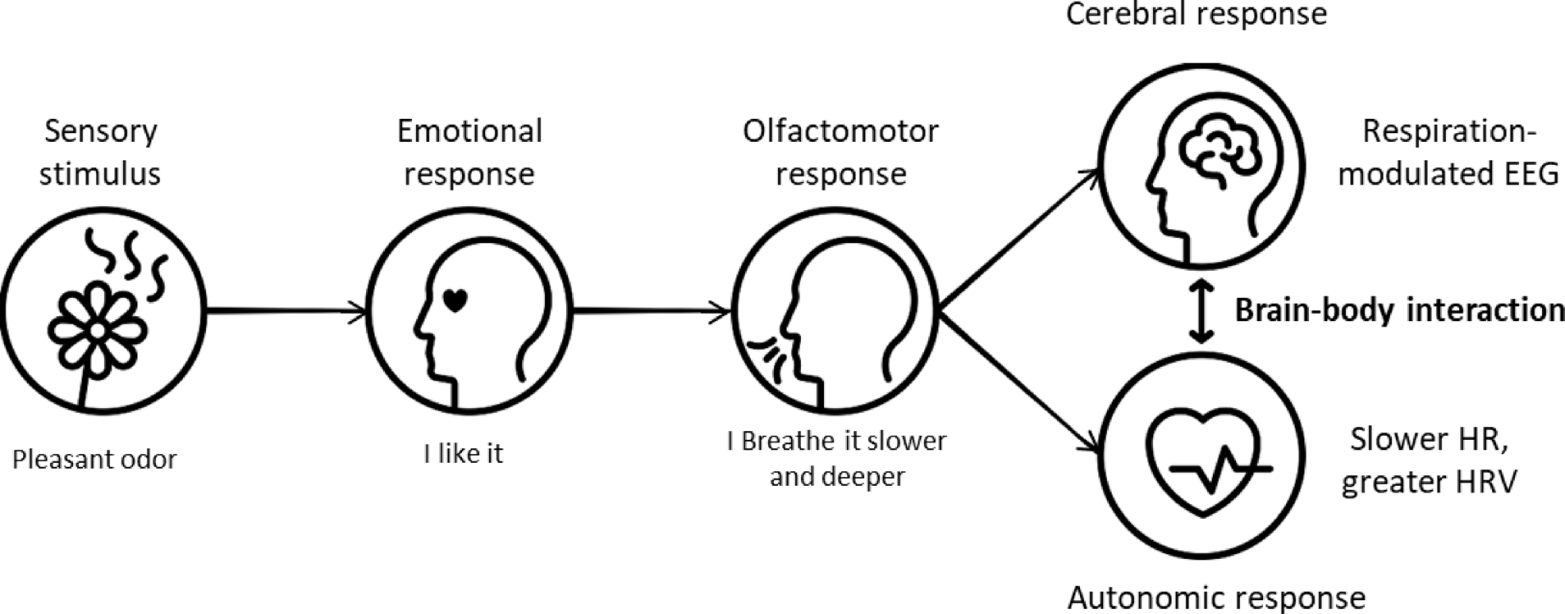
Hypothetical events sequence explaining the specific effects of pleasant odors on brain-body interaction.

Our assumption is that most of the autonomic and cerebral effects we observed are a consequence of the olfactomotor response, as illustrated in Fig. 8. Indeed, since odors are primarily experienced in terms of emotions^24,70,71^, a pleasant odor first evokes an emotional response (“I like it”) which triggers an olfactomotor response (“I breathe it slower and deeper”), therefore eliciting both autonomic (heart rate decrease) and brain (respiration-related EEG activity) responses. That could explain the large temporo-parieto-central network of respiration-related EEG activity we observed under the pleasant odor condition. This network could testify to the involvement of the premotor, motor and somatosensory cortices, which are involved in volitional and attentive breathing^8^. It would be interesting to use other methods, such as MEG or intracerebral EEG, to highlight the probable involvement of regions linked to emotions or interoception, which are not directly recordable by EEG.

### Music pleasantness is not a relevant parameter for inducing coupling between body and brain rhythms

In contrast to odor, we observed that pleasant music, even with slow tempo, accelerated breathing rate and had no effect on cardiac activity. Music increased the subjective feeling of arousal more than pleasant odors did. This suggests that pleasant music tends to induce a physiological arousal, while pleasant odors induce a physiological appeasement. This confirms what was described before, that, compared with silence, music increases heart and respiratory frequencies, which are higher during pleasant than unpleasant music listening^72,73^. However, other studies reported that certain types of music, especially those with slow tempi, slow down both cardiac and breathing rates^74^. Recently, Baccarani et al.^75^ reported that, during a stress recovery period, music elicited cardiac modifications. This discrepancy with our results could be due to different factors. For example, previous work found differential effects of tempi on the physiology in musician vs. non-musician participants^74^. Among plausible influencing factors, the listening context is probably one of the most important contributors^76^.

### Pleasant odors do not significantly improve the subjective feeling of relaxation: a conflicting effect

We observed that, contrary to some previous findings, neither odors nor music significantly altered the subjective perception of relaxation, despite a tendency to improve it^24,26,77–79^. Given the extensive literature on the subject^1,3,80,81^, we expected that a slower respiratory rate, such as that evoked by odors, would also be associated with a greater sense of relaxation. However, in our experiment, the subjective relaxation measure did not correlate with what is generally considered as objective markers, i.e. decrease in breathing rate and increase in HRV^1^. Zaccaro et al. have previously noted “limited evidence” regarding the relationship between physiological and psychological outcomes in the context of slow breathing techniques and anxiety^1^.

One possible reason for this lack of effect could be that participants were already in a relatively calm state upon arrival, as indicated by the relatively high baseline level of subjective relaxation (~ 0.65). Another possibility is that our single-item question about the present feeling of relaxation may have been insufficient to capture such a complex feeling, and a more in-depth questionnaire could provide a more reliable measurement and reduce the variability inherent in assessing this state.

Most importantly, behavioral and physiological responses to odors can vary enormously among participants depending on many factors such as culture, previous experiences, mood, expectation, and many others (for a review, see^30^. Several research groups have emphasized the importance of the notion of “personally pleasant” when using odorant stimuli^24,26,71,78^. We prioritized this parameter in our study, which we consider to be one of its strengths. However, this approach could also present a limitation, as, while we controlled for the high pleasantness of the odor, the arousal parameter was left variable. In such conditions, a pleasant odor could vary from very slightly arousing to highly arousing. Since arousal has significant impact on the autonomic nervous system^25,82–85^, it is not surprising that we observed no increase in the subjective feeling of relaxation, despite a notable autonomic response.

## Conclusion

We demonstrated that the positive emotion evoked by a personally pleasant odor, through the olfactomotor response it triggers, is capable of synchronizing brain and body rhythms. Such coupling, however, cannot be achieved by listening to pleasant music, at least in our experimental conditions. Given its non-invasive nature and ease of application, the use of odors (e.g., scents, perfumes, essential oils) presents a promising approach to improve both bodily and brain state, and, consequently, mental well-being.

Acknowledgments.

## Supplementary Information

Below is the link to the electronic supplementary material.

Supplementary Material 1

## Data Availability

All data are available upon reasonable requests from Valentin Ghibaudo ([valentin.ghibaudo@inserm.fr] (mailto: valentin.ghibaudo@inserm.fr)).

## Abbreviations

BL: Baseline
BMRQ: Barcelona Music Reward Questionnaire
CPP: Comité de Protection des Personnes (Ethics Committee)
ECG: Electrocardiogram
EEG: Electroencephalogram
HRV: Heart rate variability
ICA: Independent component analysis
MAD: Median absolute deviation
OAS: Odor Awareness Scale
RSA: Respiratory sinus arrhythmia
ANS: Autonomic nervous system

## Code availability

All analysis scripts used for this study are available at this link: https://github.com/ValentinGhibaudo/Scripts_Odor_Music_Brain_Body.
