# Supplementary material for: Pleasant odors specifically promote a soothing autonomic response and brain–body coupling through respiratory modulation
Source: Sci Rep. 2025 Oct 17;15:36417. doi: 10.1038/s41598-025-20422-x (PMC12534574; doi:10.1038/s41598-025-20422-x)
Supplement: Supplementary file 1 — Supplementary Material 1 [file 41598_2025_20422_MOESM1_ESM.docx]

**Supplemental information**

**Supplementary Table 1**: Dilution table of odorant solutions. Odorants are designed by Jean-Charles Sommerard (Sevessence)

| **Odorant** | **Dilution (% stock solution)** |
| --- | --- |
| Blue ocean | 50 |
| Cotton flower | 20 |
| Olive leaf | 50 |
| Oxygen | 20 |
| Peach Lavender | 20 |
| Rose | 50 |
| Spiced orange blossom | 20 |
| Spiced wood | 10 |
| Spring floral | 50 |
| Vanilla | 75 |

**Supplementary Table 2: Categories of music evaluated by participants**

| **Music genre** | **Reference** | **Average tempo (bpm)** |
| --- | --- | --- |
| Classical | Sir Neville Marriner - Was mir behagt, ist nur die muntre Jagd, BWV 208: IX. Schafe können sicher weiden (Orch. Marriner) (1713) | 55 |
| Electronic | Janji - Heroes Tonight (2015) | 128 |
| French variety | Isabelle Boulay – Parle-moi (2000) | 81 |
| Hard rock | AC/DC - Kick You When You Down (2020) | 110 |
| Jazz | Herbie Hancock - Speak Like a Child (1968) | 122 |
| Metal | Aephanemer - Path Of The Wolf (2019) | 134 |
| Pop | Miley Cyrus - Adore You (2013) | 60 |
| Rap #1 | Freeze Corleone - Freeze Raël (2020) | 68 |
| Rap #2 | Luv Resval - AZNVR (2023) | 108 |
| Raga | Zia Mohiuddin Dagar – Dhrupad, Raga Yaman (1991) | 56 |

bpm: beats per minute.


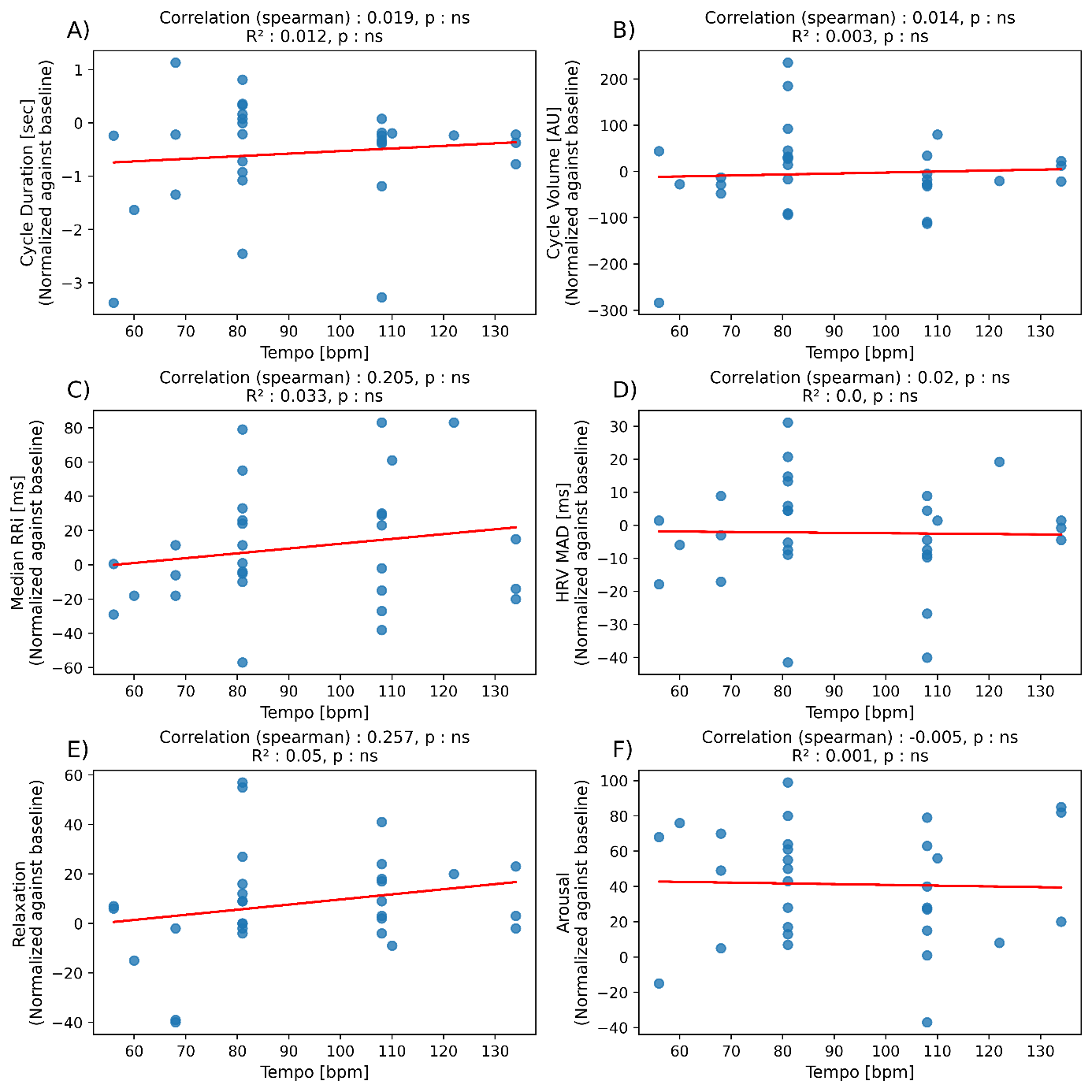


Figure S1: Effects of music tempi on physiological and subjective markers of relaxation (**A)** Respiratory cycle duration, (**B)** Respiratory total cycle volume, (**C)** Median value of RR interval, (**D)** Median Absolute Deviation of RR interval (HRV MAD), (**E)** Subjective relaxation according to music tempo, and (**F)** Subjective arousal. Each participant listened to music with a specific tempo, as indicated on the x-axis. Values for each metrics are presented on the y-axis. Data from the music condition were normalized by subtracting baseline data (music data – baseline data). The red line represents the regression line. Statistical results are presented in the title of each subplot, including the Spearman correlation coefficient, regression coefficient, and *p*-values interpretation of (ns: non-significant, *: *p* < 0.05).


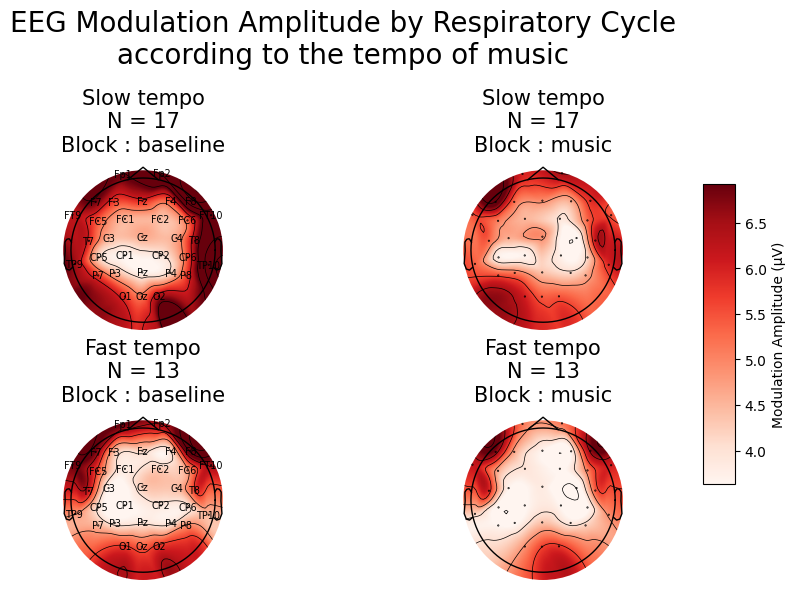


Figure S2: Effects of music tempi on EEG modulation by respiratory cycle. The amplitude of EEG modulation by respiratory phase was measured for each participant and electrode, under both baseline (left) and music (right) conditions. Each participant listened to music with a specific tempo, which was classified into two categories: Slow (<90 bpm; n = 17) and Fast (>90 bpm; n = 13). The averaged values are presented topographically for both the baseline and music conditions (left and right panels respectively), split by the tempo category, upper panels for S*low tempo, lower panels for Fast tempo). Cluster-based permutation tests comparing the music condition against baseline for each tempo category did not reveal a significant effect of tempo on EEG modulation.
